# Longitudinal Coadaptation of Older Adults With Wearables and Voice-Activated Virtual Assistants: Scoping Review

**DOI:** 10.2196/57258

**Published:** 2024-08-07

**Authors:** Kristina Marie Kokorelias, Alisa Grigorovich, Maurita T Harris, Umair Rehman, Louise Ritchie, AnneMarie M Levy, Kerstin Denecke, Josephine McMurray

**Affiliations:** 1 Division of Geriatric Medicine Department of Medicine Sinai Health System and University Health Network Toronto, ON Canada; 2 KITE Research Institute Toronto Rehabilitation Institute University Health Network Toronto, ON Canada; 3 Recreation and Leisure Studies Brock University St Catherines, ON Canada; 4 User Experience Design Wilfrid Laurier University Waterloo, ON Canada; 5 Department of Computer Science University of Western Ontario London, ON Canada; 6 Alzheimer Scotland Centre for Policy and Practice University of West Scotland Scotland United Kingdom; 7 Lazaridis School of Business and Economics/Community Health Wilfrid Laurier University Brantford, ON Canada; 8 Institute for Medical Informatics Bern University of Applied Sciences Bern Switzerland

**Keywords:** older adults, coadaptation, voice recognition, virtual assistant, wearable, artificial intelligence, smart-assistive technology, scoping review, review methods, review methodology, knowledge synthesis, synthesis, scoping, older adult, gerontechnology, technology, smart technology, smart technologies, smart, geriatrics, older people, geriatric, scoping literature review, protocol, Internet of Things, IoT, aging, PRISMA-ScR, Preferred Reporting Items for Systematic Reviews and Meta-Analyses Extension for Scoping Reviews, user-centered design, design, user centered, mobile phone

## Abstract

**Background:**

The integration of smart technologies, including wearables and voice-activated devices, is increasingly recognized for enhancing the independence and well-being of older adults. However, the long-term dynamics of their use and the coadaptation process with older adults remain poorly understood. This scoping review explores how interactions between older adults and smart technologies evolve over time to improve both user experience and technology utility.

**Objective:**

This review synthesizes existing research on the coadaptation between older adults and smart technologies, focusing on longitudinal changes in use patterns, the effectiveness of technological adaptations, and the implications for future technology development and deployment to improve user experiences.

**Methods:**

Following the Joanna Briggs Institute Reviewer’s Manual and PRISMA-ScR (Preferred Reporting Items for Systematic Reviews and Meta-Analyses Extension for Scoping Reviews) guidelines, this scoping review examined peer-reviewed papers from databases including Ovid MEDLINE, Ovid Embase, PEDro, Ovid PsycINFO, and EBSCO CINAHL from the year 2000 to August 28, 2023, and included forward and backward searches. The search was updated on March 1, 2024. Empirical studies were included if they involved (1) individuals aged 55 years or older living independently and (2) focused on interactions and adaptations between older adults and wearables and voice-activated virtual assistants in interventions for a minimum period of 8 weeks. Data extraction was informed by the selection and optimization with compensation framework and the sex- and gender-based analysis plus theoretical framework and used a directed content analysis approach.

**Results:**

The search yielded 16,143 papers. Following title and abstract screening and a full-text review, 5 papers met the inclusion criteria. Study populations were mostly female participants and aged 73-83 years from the United States and engaged with voice-activated virtual assistants accessed through smart speakers and wearables. Users frequently used simple commands related to music and weather, integrating devices into daily routines. However, communication barriers often led to frustration due to devices’ inability to recognize cues or provide personalized responses. The findings suggest that while older adults can integrate smart technologies into their lives, a lack of customization and user-friendly interfaces hinder long-term adoption and satisfaction. The studies highlight the need for technology to be further developed so they can better meet this demographic’s evolving needs and call for research addressing small sample sizes and limited diversity.

**Conclusions:**

Our findings highlight a critical need for continued research into the dynamic and reciprocal relationship between smart technologies and older adults over time. Future studies should focus on more diverse populations and extend monitoring periods to provide deeper insights into the coadaptation process. Insights gained from this review are vital for informing the development of more intuitive, user-centric smart technology solutions to better support the aging population in maintaining independence and enhancing their quality of life.

**International Registered Report Identifier (IRRID):**

RR2-10.2196/51129

## Introduction

### Background

Technology has revolutionized our lifestyles from communication methods and social interactions to mobility and self-care. The integration of artificial intelligence (AI) into household devices, such as built-in voice-activated virtual assistants, epitomizes the “Internet of Things” (IoT) concept, where devices are interconnected over the internet allowing for remote monitoring and control [1]. Gigli and Koo [2] described the IoT concept as an “ongoing movement to consolidate all resources globally into a shared infrastructure.” This technological integration is particularly significant for individuals aged 60 years and older, whose numbers are expected to rise significantly in the coming decades [3]. Ambient-assisted living (AAL) technologies, which form a core part of IoT applications tailored to personal care, enhance the quality of life and support the independence of older adults within their living environments [4,5]. By leveraging IoT capabilities, AAL technologies can provide continuous assistance and monitoring, thereby enhancing the safety, independence, and well-being of older adults and supporting the concept of aging in place [4,5].

Aging in place is crucial for enabling older adults to stay socially connected to the places where they live and feel comfortable and competent [6-9], thus potentially reduce the demand for institutional care, alleviating the burden on health care systems [10-14]. Smart technologies facilitate access to the benefits of the internet and video calls for social interaction and access to care and are also equipped with sensors and automation systems [15] that enhance self-management of chronic conditions and adapt living spaces to accommodate the changing needs of older adults [16-20]. For example, such technologies can enable remote or automated control of various features in the home, including lighting, temperature, and use of multimedia, as well as provide automated fall detection, all of which can mitigate potential hazards and accidents in the home [21,22]. These technologies are believed to contribute to high-quality care standards in the care of older adults [23,24].

While the benefits and potential applications of IoT technologies to support older adults aging in place are well known [25,26], particularly in areas such as health management and emergency readiness [27], there is a gap in the scientific literature regarding the long-term interaction (eg, beyond the first few weeks of use) between smart technologies and this demographic [15]. Cross-sectional studies have provided valuable insights into older adults’ intentions to use smart technologies [28] and some initial aspects of coadaptation [29]. However, these studies typically capture a single point in time and fail to reflect the evolving nature of use and adaptation. Consequently, there is a scarcity of research that systematically explores the dynamic, ongoing process of coadaptation where behaviors or actions of older adults change beyond becoming familiar with the technology, and smart technologies using automated, algorithm-driven adaptations adjust to user behaviors and preferences in real time [30] and continuously adjust to each other over extended periods [31].

Ongoing interaction is essential to fully understand the potential and challenges of smart technologies in real-world settings. This need for continuous engagement, a process referred to as coadaptation, has been noted as a limitation in previous reviews [28]. Effective integration of technologies relies not only on their technical capabilities but also on a robust process of coadaptation, the dynamic and reciprocal adjustments between older adults and smart technologies that enhance functionality, usability, and overall user experience [32]. This ongoing interaction ensures that the technologies evolve to meet the specific needs of older adults while the users adapt their behaviors to maximize the benefits of the technology. For example, voice-activated devices may adapt to recognize and understand the speech patterns of their users over time, improving interaction quality [33]. Such interactions ensure that technologies evolve to meet the specific needs of older adults, allowing users to optimize use and the benefits of the technology. However, this is an emerging domain as AI is increasingly incorporated into everyday consumer products. The gap we have identified underscores the need for ongoing research to understand and improve how technologies can better adapt to user preferences over time. This is crucial for improving the independence, user experience, and sustained technology adoption of older adults.

### Objectives of the Scoping Review

This scoping review addresses the research gap by examining existing literature on the interaction between smart technologies and older adults over time. It focuses on wearables and voice-activated virtual assistants, exploring how these interactions adapt to enhance user experiences and the benefits derived from these technologies. We chose to focus on wearables and voice-activated devices as they are some of the most common smart technologies used by older adults, they are the key demographic in our study, and they offer convenience and accessibility by often interacting with other smart home technologies [34-36]. This study aims to address several critical questions regarding the nature of coadaptation between smart technologies and older adults, the outcomes of this process, strategies used by older adults to adapt, and the methodological approaches in the existing literature. Specifically, this review seeks to address the following questions:

1. What is the extent and nature of the existing scientific literature exploring the coadaptation between smart technologies and older adults, and how do older adults and technology coevolve over time to enhance older adults’ experience with the technology?
2. What have been the outcomes of the coadaption between older adults and smart technology?
  - What specific outcome measures have been used in studies investigating the experiences of older adults in terms of coadapting with technology over time?
3. What specific strategies or approaches have participants used to adapt to the changing circumstances, challenges, or opportunities in their environment, and what process do they follow when making adaptive decisions?
4. What are the key characteristics of older adult participants who have been involved in studies examining the coadaptation between smart technologies and older adults?
5. What methodological strengths, limitations, and recommendations have been documented in the literature regarding the exploration of coadaptation between smart technologies and older adults?
6. What research models and theories contribute to the conceptualization of coadaptation?

By answering these questions, this study aims to support further research on tailoring AI-driven technologies to the specific needs of older adults, developing more user-friendly technologies, easing caregiver burden, and formulating policies promoting independent living.

## Methods

### Design

To address the diverse range of research questions, we used a scoping review methodology recommended for studies aiming to comprehensively map the literature on complex or heterogeneous topics [37]. This scoping review followed the procedures outlined in the Joanna Briggs Institute manual for conducting scoping reviews [38] and reported in accordance with the PRISMA-ScR (Preferred Reporting Items for Systematic Reviews and Meta-Analyses Extension for Scoping Reviews) guidelines (Multimedia Appendix 1) [39]. Key deviations from the initially registered protocol [15] include changes in the inclusion criteria related to the duration of technology use and expansion of the search terms and databases to ensure comprehensive literature coverage. These adjustments were necessary to address the rapid evolution of technology and its adoption, ensuring the relevance and comprehensiveness of our review.

### Eligibility Criteria

We included peer-reviewed empirical studies, dissertations, and conference proceedings that were published in English since 2000 to include the most recent studies and technologies pertinent to the topic under review [15]. The year 2000 was chosen as a starting point for the literature search to focus on the most recent 2 decades of advancements and their application in supporting older adults, ensuring the review captures relevant and contemporary studies. The turn of the millennium saw significant advancements in computing technology, such as faster processors, increased memory capacity, and improved capabilities [40,41]. Studies had to include individuals aged 55 years or older living independently in community-based settings (not institutional care) and explore the interactions, coadaptation, experiences, and outcomes among older adults and smart technologies, specifically wearables and voice-activated virtual assistants. The coadaptive technology had to be automated, algorithm-driven, and adjusting to user behaviors and preferences in real time to be included in the study—a criterion that we believed accommodated the diverse needs of the older adult users who are more likely to face challenges associated with lower technical proficiency [42] and declining physical and cognitive functions [43]. In line with other reviews on technology and older adults, we modified the age threshold for older adults to 55 years or older to ensure inclusivity and comprehensiveness to capture early experiences of aging and technological interaction, thereby providing a comprehensive view of the aging spectrum relevant to technology use [44].

Our review highlights aspects such as quality of life, well-being, social connectedness, independence, and overall user experience over a minimum average period of 8 (SD 2) weeks or more . Based on our preliminary analysis and expert consultations, a minimum duration of an average of >8 (SD 2) weeks was identified as reasonable for observing meaningful coadaptation between older adults and smart technologies. This duration is considered sufficient to allow for user familiarization, routine integration, and initial feedback cycles essential for coadaptive processes to become apparent and is supported by literature on behavioral habituation and technology adoption, which suggest that several weeks (more than a month) are required for users to adapt to and integrate new technologies into their daily lives [31,45,46]. We excluded studies that focused on other types of technologies or forms of literature.

Three reviewers (KMK, AG, and JM) further refined the a priori eligibility criteria. In line with the iterative nature of conducting scoping reviews [47], the original minimum study duration requirement of 6 months or more [15] was adjusted to an average of >8 weeks to accommodate the limited volume of relevant literature. We had originally selected a 6-month duration based on our knowledge of at least 1 paper that conducted a year-long study, considering 6 months to be a generous timeframe; this paper is included in our review.

### Search Strategy

The primary and senior authors (KMK and JM), in collaboration with an experienced medical information specialist, iteratively developed search strategies with feedback from all authors (Multimedia Appendix 2). The keywords for the review included age-related terms (“aged,” “seniors,” “older adults,” “elderly,” “people with disabilities,” and “cognitive and physical disabilities”), technological terms (“wearable electronic devices,” “voice recognition,” “artificial intelligence,” “smart technology,” “smart assistive technology,” “virtual reality,” and “ambient intelligence”), and interaction-related terms (“coadaptation,” “double-loop learning,” “coevolution,” “human-computer interface,” “human-environment interaction,” “interaction design,” “personalization,” “customization,” “tinkering,” “crafting,” “redesign,” “modification,” “sensemaking,” “hacking,” “accommodation,” “mutual adaptation,” “symbiotic evolution,” “reciprocal adjustment,” “interactive iteration,” “user-technology synergy,” “dynamic interface refinement,” “adaptive coevolution,”” concurrent learning,” and “bidirectional refinement”).

Peer review of the Ovid MEDLINE search strategy was conducted using the PRESS (Peer Review of Electronic Search Strategies) checklist by an information specialist from another institution [48]. Adjustments in vocabulary and syntax were then applied across multiple electronic databases (Ovid Embase, PEDro, Ovid PsycINFO, EBSCO CINAHL, IEEE Xplore, Web of Science, the Cochrane Library, Scopus, and Global Index Medicus). The initial search was carried out on August 28, 2023, aimed at identifying English-language publications from 2000 onward, complemented by scanning reference lists, forward and backward searching, and seeking expert recommendations to ensure comprehensive coverage. EndNote (version 9.3.3; Clarivate Analytics) facilitated the download and deduplication of retrieved records [49]. The search was updated by the same information specialist on March 1, 2024, but no additional papers were included.

### Study Selection

Deduplicated records were imported into Covidence software (Veritas Health Innovation) [50]. Titles and abstracts were screened by at least 2 reviewers from the full research team followed by a full-text screening. In our scoping review, we applied a hierarchical exclusion process prioritizing the wrong study design (eg, commentaries and reviews), incorrect technology, a duration of less than 8 weeks, and the absence of coadaptation. This meant some papers were excluded for multiple reasons. These exclusions were reported from highest to lowest frequency in our PRISMA-ScR flow diagram. Discrepancies during study selection were resolved through discussions among the research team, ensuring that decisions were made collectively and based on a consensus, which adds to the credibility and reliability of the selection process. One reviewer (KMK) conducted the hand screening consisting of the forward and backward screening and the review of reference lists. All team members reviewed potentially included papers to confirm the selection.

### Data Extraction and Analysis

Using Covidence, a data extraction form was iteratively developed and pilot-tested by reviewers (KMK, AG, and JM), facilitating the extraction of study characteristics; participant demographics such as sex and gender; underlying research models, theories, and frameworks; study findings; implications for the technology coadaptation process; and any identified study quality or limitations. Data were extracted by 1 team member (KMK), with uncertainties discussed and resolved among the research team.

Data analysis involved a descriptive review of included papers detailing study characteristics, followed by a content analysis by 3 reviewers (KMK, AG, and JM), with a specific focus on changes in user behavior patterns over time, interaction modifications, and instances where both users and technologies engaged in reciprocal feedback loops resulting in iterative adaptations.

## Results

### Overview

The database literature searches yielded 16,143 unique records that were screened at the title and abstract phase. A total of 94 papers were screened at the full-text stage, and a total of 5 papers were included. To improve transparency and to provide insights into our rigorous selection process, Multimedia Appendix 3 summarizes the primary reasons for excluding the 89 papers that had an insufficient focus on the interactive and mutual adaptation between older adults and technologies. This table aims to further clarify our stringent criteria for coadaptation, which necessitates observing changes and adjustments over time that significantly impact user experience and technology efficacy.

**Figure 1:**
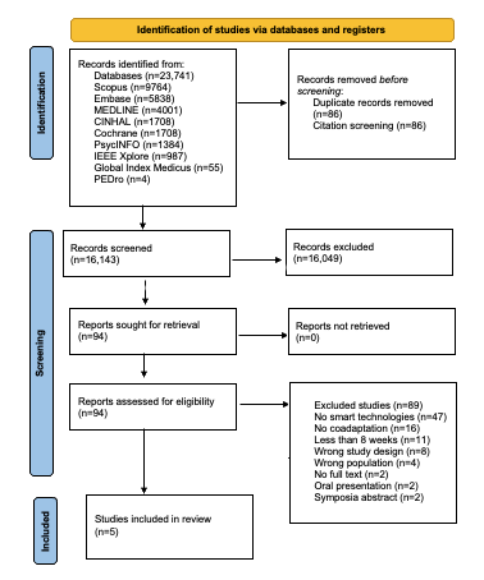
PRISMA flow diagram.

The finding that only 5 papers met the inclusion criteria highlights a significant gap in research on the long-term coadaptation between older adults and smart technologies, underscoring the need for further investigation in this emerging field. One of these papers was found during the hand search. Figure 1 [51] presents the PRISMA (Preferred Reporting Items for Systematic Reviews and Meta-Analyses) flow diagram illustrating the paper selection process.

### Main Characteristics of the Included Papers

All studies were published between 2021 and 2023. In total, 2 studies were mixed methods [52,53], 2 were qualitative [54,55], and 1 was quasi-experimental [56]. The qualitative data collection methods in these papers included observations [52,55] and interviews [52-54] to understand the experiences, challenges, and interactions of older adults with conversational agents (CAs) and virtual assistants. Three studies kept interaction logs to capture the use of participants [53], including interactions initiated by users through voice commands [52,54]. Another study used a quantitative survey at recruitment and after 2 weeks of use [53].

All but 1 study were conducted in the United States; the remaining study was conducted in the United Kingdom [53]. Three studies involved data collection in people’s homes [52,54,55]. Two studies involved data collected at health care facilities [53,56]. Table 1 presents an overview of the included papers.

**Table 1:** Characteristics of included peer-reviewed studies.

Author (year), country, and design	Objective	Methods for data collection and number of participants	Length of intervention	Results	Limitations	Recommendations
Cuadra et al (2023) [55], United States, and qualitative	The objective of this study was to explore the experiences and perceptions of older adults who have become users of voice-activated technologies, specifically through the use of a VFAI^a^. The study focuses on understanding how these individuals interact with the VFAI in various contexts, including health data reporting and positive reminiscing, and how their lived experiences influence their use patterns and perceptions of the technology.	The researchers conducted interviews with 5 participants, reviewed use logs, and provided support to participants to closely observe their interactions with the VFAI over a period of time.	5.9-12.6 weeks (average 8.64 weeks)	The interactions with design probes for health data reporting and positive reminiscing were generally positive, with participants appreciating the value suggested by the probes. However, there were challenges with expectations regarding Alexa’s ability to interpret open-ended dialogue and support ambiguity. Participants felt that Alexa did not judge them and provided a safe space for interaction. Overall, participants perceived Alexa as a companion and found the display helpful for voice-first interactions. Challenges included skepticism about health data reporting, hesitance before using the probes, and usability issues such as communication breakdowns. Addressing these challenges could enhance the user experience and increase the value of VFAIs for supporting aging in place.	The study has limitations including nonfunctional design probes, a small sample size of 5 participants in an urban US setting, and the availability of technical support, which may not reflect real-world scenarios for older adults. Additionally, participants were relatively healthy, potentially limiting generalizability, and privacy risks associated with VFAI use require careful consideration in future research.	The study recommends leveraging virtual personal assistants (VFAI) to enhance continuity of care for older adults in their homes. VFAIs can support management continuity by providing consistent health management and reminders for tasks like medication intake and exercise. They can facilitate relational continuity by fostering ongoing therapeutic relationships with users, offering emotional support, and maintaining a nonjudgmental and always available presence. Additionally, VFAIs can promote informational continuity by collecting and using robust information about users’ preferences, health history, and context to tailor interactions and support personalized care plans effectively.
Gvozdanovi et al (2022) [53], United Kingdom, and mixed methods	The objective was to assess the feasibility of integrating Vinehealth into brain tumor care, evaluating its capacity to gather real-world and ePRO^b^ data and measure subjective improvements in care.	The study adopted a mixed methodology IDEAL^c^ stage 1 design and was conducted at a single tertiary care center. In total, 6 patients were enrolled in the study, of which 4 actively participated by downloading and engaging with the mHealth^d^ app throughout the 12-week duration. The primary focus was on collecting real-world and ePRO data through Vinehealth over the study period. Additionally, qualitative feedback was gathered through mixed methodology surveys and semistructured interviews conducted at recruitment and after 2 weeks. This approach allowed for a comprehensive assessment of the feasibility of integrating Vinehealth into brain tumor care as well as evaluating its effectiveness in gathering relevant data and subjective improvements in patient care.	12 weeks	A total of 565 data points were recorded, encompassing various aspects such as symptoms, activity levels, well-being, and medication use. Technical difficulties affected the completion rates of the EORTC^e^ QLQ-BN20 and EQ-5D-5L surveys, with rates of 54% and 46%, respectively. However, when ePROs were used, completion rates reached 100%. Participants expressed a desire for more content specific to brain cancer tumors. Despite challenges, all participants endorsed the app and perceived it as enhancing their care.	First, being a proof-of-concept study (IDEAL stage 1), the small sample size precluded the inclusion of a comparator group. Future stages (2 and 3) as per the IDEAL framework would necessitate a larger sample size for formal outcome assessment. While the aim was to onboard all patients before surgery, logistical challenges during the COVID-19 pandemic disrupted this plan. The withdrawal of 2 of 6 participants and the pragmatic recruitment approach highlight the need for a more robust recruitment strategy and a better understanding of withdrawal reasons in future studies. Technical issues and retention rates limited the availability of ePRO data, which could be addressed in subsequent iterations through improved technology and longer follow-up periods with a larger cohort. Additionally, future studies should involve the multidisciplinary team, facilitated by an HCP^f^ dashboard for real-time data review and collection of HCP feedback.	—^g^
Kim and Choudhury (2021) [54], United States, and qualitative	This study aims to gather empirical evidence on how the experiences of older adults with a virtual assistant change over time, from novice to experienced users.	This study used a 3-phase interview protocol to investigate older adults’ perceptions, challenges, coping strategies, and use patterns with a virtual assistant over a 16-week period. Participants were introduced to a Google Home mini during the first interview, followed by 8 biweekly follow-up interviews. The study also collected device use logs from Google’s activity history repository to complement participants’ perceptions with actual interaction patterns. 12 participants	16 weeks	Participants used the virtual assistant primarily for tasks such as playing music, searching for information, making casual conversations, and checking the time and weather. Despite initial challenges, including unfamiliarity with the device and functional errors, participants gradually developed competence and resilience in interacting with the voice-activated virtual assistant. Over time, they appreciated the simplicity and ease of use, convenience of operating without physical interaction, and benefits such as not worrying about making mistakes and building digital companionship.	The study’s limitations include potential biases due to convenience sampling from older adult–living communities, the exclusion of older adults with hearing impairments, the lack of comparative studies across different age groups, and the inability to analyze use patterns quantitatively due to data collection through a single Google account.	The recommendations include enriching the conversational capabilities of virtual assistants to enhance user experiences, supporting a learning phase for novice users, and revisiting form factors to align with user expectations and affordances, particularly for older adults who may experience difficulty with new technologies.
Kim (2023) [56], United States, and quantitative	The objective was to assess the impact of integrating information and communication technologies into the health care management of older individuals aged 65 years or older by providing them with the “Health Today” app and a smart speaker for 6 months, measuring changes in depression, self-efficacy, moderate-intensity exercise frequency, relative grip strength, balance, and 5-times-sit-to-stand performance through preintervention and postintervention evaluations.	166 participants, aged 65 years or older and enrolled at public health centers, were given access to the “Health Today” app and a smart speaker for 6 months to complete health care tasks. The study divided participants into 2 groups: one group received both the app and speaker, while the other only used the app. Assessments of depression, self-efficacy, frequency of moderate-intensity exercise, grip strength, balance, and 5-times-sit-to-stand performance were conducted before and after the 6-month program.	6 months	Both cohorts demonstrated improved health status and behavioral shifts during the postintervention assessment. Nevertheless, the group given smart speakers, alongside the health management app did not exhibit decreased depression, potentially attributable to the communication and music-listening features.	The study lacked control over attitudes and use patterns toward smart speakers. Follow-up studies should aim to identify and control use patterns for digital devices. Given that prior research predominantly focused on middle-aged individuals, additional studies investigating the effects of mHealth on older adults are warranted. Variables, such as grip strength and balance tests recognized as reliable indicators of frailty, should be taken into account.	—
Zubatiy et al (2023) [52], United States, and mixed methods	The objective of this research study is to investigate how older adults with MCI^h^ and their caregivers use CAs^i^ in their homes over time, with a focus on understanding the limitations of existing CAs and identifying requirements for future systems to address these limitations, without externally imposed goals or tasks.	The study conducted three 10-week deployments of Google Nest Hub devices in the homes of participants, conducting semistructured interviews to understand the impact and use of the devices. User attrition occurred during the deployments, and interaction logs were collected using Google Takeout, allowing for quantitative analysis of user interactions categorized by type and difficulty levels. The goal was to capture perspectives on the usefulness, entertainment value, and frustrations of using the devices in day-to-day life without imposing interaction targets on participants. 26 older adults and their caregivers	3×10 weeks	While both care partners and individuals with MCI interacted with the device, there was a consistent increase in use over time, particularly in the third deployment. However, challenges such as multiuser interactions, lack of personalization, and inflexibility in error recovery were identified as persistent themes across all deployments, impacting the overall user experience and effectiveness of the devices.	Limitations include the reliance on a patient population with MCI and their cognitively normal aging spouses, the lack of a true healthy control group, the imperfect metrics used to measure MCI, the limited demographic diversity of the participants, variations in training protocols, and the small sample size, necessitating further research to generalize the findings to broader populations and validate the observed trends.	The study recommendations include the need for future CA systems to prioritize flexibility over efficiency in interactions, encouraging more robust error handling and facilitating user-friendly error recovery mechanisms to minimize frustration and optimize user experience. Additionally, there is a call for enhancing personalization while balancing privacy concerns, suggesting that CA systems should learn and adapt to user preferences over time while respecting user privacy and autonomy. Finally, the study advocates for a shift toward more proactive interactions in CA systems, particularly in multiuser scenarios, to reduce the cognitive burden on users, improve accessibility, and enhance overall usability.

^a^VFAI: voice-first ambient interface.

^b^ePRO: electronic patient-recorded outcome.

^c^IDEAL: Idea, Developments, Exploration, Assessment and Long-term follow-up

^d^mHealth: mobile health.

^e^EORTC: European Organization for Research and Treatment of Cancer.

^f^HCP: health care professional.

^g^Not available.

^h^MCI: mild cognitive impairment.

^i^CA: conversational agent.

### Technology Use and Outcomes

Three studies involved older adults using a voice-activated virtual assistant on either a Google or Amazon smart speaker [52,54,55]. Another study used an unnamed smart speaker [56]. One study involved wearables [53]. Two studies involved health apps [53,56,57]. Study intervention periods ranged from 5.9 [55] to 16 weeks [54]. Across all the studies, the use of the technology was consistently maintained [52]. The majority of interactions involved simple user-initiated cues to the technology [52,53], and the mean use frequency averaged 1.8 times daily [54]. The most frequent use of the voice-activated virtual assistant involved playing music, general information searches (eg, weather), casual conversations, and setting reminders or alarms [54]. One study also noted that use patterns revealed use peaks at 9 PM and 7 AM, aligning with bedtime and waking hours [54]. A daytime surge between 1 and 4 PM lacked a defined use pattern, potentially indicating leisure time between meals [54]. Participants tended to value the simplicity and ease of interaction of voice-activated technologies, particularly the fact that they resulted in hands-free convenience [54-56]. In 1 study using wearables, participants appreciated the seamless integration of apps with Google Fit and Apple Health, monitoring vital signs including heart rate and blood pressure, with particular attention to activity levels due to perioperative fatigue in individuals with brain cancer [53].

### Reported Characteristics of Study Participants

Sample sizes ranged from 5 [54,55] to 166 participants [56]. All but 1 study reported the average age of participants. One study reported a range of 45-69 years [53]. The average age of the older adult participants ranged from 73 [55] to 83.8 years [54]. Four (80%) of the 5 studies included mostly female participants [53-56], while another included an equal number of both male and female participants [52]. None of the studies included gender-diverse people (ie, nonbinary).

In regard to health status, one study targeted older adults diagnosed with mild cognitive impairment (MCI; n=13 male and n=13 female) along with their 26 respective care partners (n=10 male, n=16 female; average age 65.7 years) [52]. Caregivers were a spouse or partner or adult child [52]. Diagnosis of MCI had to be validated by a neurologist through a standard set of neuropsychological tests, including the Montreal Cognitive Assessment scores [52]. The focus was on understanding their interactions with CAs in caregiving networks. Another study targeted individuals with brain cancer [53]. In another study with 12 participants, 2 (17%) participants were reported as wearing a hearing aid, and 3 (25%) participants used a wheelchair due to various joint issues [54].

Technology access and comfort were reported in all of the studies. In 1 study, nearly all participants had previously engaged with a CA at some point, although not regularly [52]. Two studies recruited novice users of voice-activated virtual assistants [54,55]. One of these 2 studies indicated that participants had to have no prior experience with a voice-activated virtual assistant [54]. Two studies noted that participants had some familiarity with computers, tablets, and smartphones [54,55]. One study did not comment on computer or smartphone use or experience [52]. In total, 4 (33%) out of 12 participants mentioned having encountered a smart speaker in their children’s homes without personal use [54]. Of these, over half of the participants (7/12, 58%) owned a tablet, and all (12/12, 100%) confirmed regular use of computers for tasks such as information searches and email. Participants in 2 studies were observed interacting with multimodal voice-activated virtual assistants in public settings to understand the challenges faced by this demographic [55,58] and their progression from novice to more experienced users [54].

The authors seldom reported on racial, ethnic, cultural, linguistic, educational, or socioeconomic characteristics of the study participants. One study did not reveal specific characteristics about their participants other than identifying their gender, age, and health concerns [54]. Participants in another study were only identified in the limitations section as being a group that reflected “largely White, upper-middle-class households” [52]. None of the studies specified or included information regarding the socioeconomic (income), education, sexual orientation, or religious affiliation of their participants.

### Theories, Models, and Frameworks Used During Study Designs

Only 1 study was informed by older adult learning theory [52]. Older adult learning theory provided a framework for interpreting older adults’ interactions with technology by emphasizing self-directed learning, relevance to life experiences, and problem-solving orientation [52]. This study posited that enhancing older adults’ successful use and acceptance of technology may benefit from diverse self-directed learning approaches, including trial-and-error, errorless learning, repetitive practice, and tailored training materials specifically aimed at this demographic, and involving members of their social support network in the learning process [52]. By contrast, the authors noted that commercial systems for CAs are often marketed as “intuitive” and seldom incorporated these or other types of learning strategies and materials to assist new users [52]. This same study used their prior work to categorize the interactions using an organizational framework by their types, such as asking for weather updates or playing music, and by levels of difficulty [52]. The authors grouped all recorded interactions into 3 broader categories: “out of the box” interactions lacked personalized or account-specific qualities, like inquiring about the weather or setting timers (level 1); personalized interactions, such as setting reminders, managing calendars, or setting alarms (level 2); and interactions requiring access to a third party “Google Action,” such as playing games or controlling home automation (level 3) [52].

### How Older Adults and Technologies Coadapt or Coevolve

Papers described the distinctions and intricacies involved in tailoring voice-activated virtual assistants’ interactions to the needs and capacities of older adults in the future to enhance use and support long-term adoption [54]. Older adult participants formed emotional connections with their voice-activated virtual assistants, seeing them as companions or friends. This relational continuity could potentially increase compliance with health-related tasks [55]. The technologies demonstrated the potential to enable management, relational, and informational continuity of care by providing consistent health management support and retaining knowledge of the users’ health histories and preferences, which may enhance users’ trust in these technologies [55]. Participants in the included studies often engaged in problem-solving to enhance their use of technology, with some noting that errors were part of their learning process [54,56]. As users began to be more familiar with technology, they exhibited resilience toward errors, attributing some to their own interaction approaches rather than solely blaming device shortcomings [54]. Individuals with brain cancer acclimated to the technology by consistently engaging with the app, inputting health information, and using the educational resources provided, showcasing a level of adaptation that surpasses mere familiarity [53]. This adaptation process was partly automated, guided by the app’s algorithms, which customize content and interventions based on the individual’s reported conditions and activities [53].

Participants also continually refined their approach to engaging with the voice-activated virtual assistants based on ongoing experiences [54]. For example, some adapted by refining their command phrasing and recognizing the inherent limitations of the technology [54]. Specifically, participants often evaluated the challenges they had with technology to engage in continuous learning and skill development to better navigate technology use [52,54]. In particular, participants developed persistent routines for using the technology [52,54] as potential solutions to challenges they had. For example, playing music before bed and checking time or weather upon waking were prevalent routines among participants [54]. Thus, over time, older adults integrated technology into their daily lives, especially around bedtime and waking hours [54].

Some participants also adapted their communication style, trialing the use of different tones and intonations with the technology; for some, such adaptation led to more successful interactions, while for others, this resulted in failure [54,55,57]. However, over time, participants began to incorporate additional words, even with mispronunciations or using alternative phrases like “Alexia” and “Alessa,” to engage the voice-activated virtual assistant. This also paraphrased commands or repeated them more clearly to enhance the device’s understanding [54,55] and adjusted their approach if their initial attempts failed [55]. When observing attempts that were successful by other individuals, participants mimicked these interactions, impacting the virtual assistant’s responses [55]. However, this created often interrupted personalized features of the technology being able to understand the primary user [55]. Attending training on the technology by care partners did not significantly inform the use of the technology [52].

### Sustained Challenges of Prolonged Technology Use

The most widely identified challenge was the result of human-machine barriers and more specifically the limited capabilities of virtual assistants to mimic human communication patterns. Communication barriers lead to frustration when the technology did not reply to participant’s replies, resulting in repeated attempts at communication [52,54]. Specifically, participants’ verbal cues aimed at signaling interaction with the technology were sometimes missed by the virtual assistant due to constraints like the need for precise word use [54,55]. Voice-activated virtual assistants with screens failed to synchronize visual prompts with participants’ responses to the technology, causing confusion and frustration [52,55]. Participants in one study had access to a technical-support contact person to resolve issues, which is not a realistic scenario for many older adults who may lack such immediate support [55]. The controlled study setting, including the availability of technical support, does not fully reflect the real-world challenges older adults might face when using such technologies independently.

### Study Strengths, Limitations, and Recommendations Noted in the Included Studies

None of the study authors explicitly mentioned the strengths of their studies. The most commonly reported limitations were related to the technology under investigation and the demographics of the participant sample. Studies had limited sample sizes and lacked participant diversity. This limitation underscores the need for future research to engage a broader and more representative sample of older adults, considering variables such as geographic location, culture, or socioeconomic status [52,54,55]. Moreover, most participants were relatively healthy; thus, the use of design probes for health data reporting could be challenging for those who are more vulnerable or less technology-savvy, highlighting the need for simpler, more intuitive interfaces [55].

Studies also primarily took place in urban environments, potentially restricting the relevance of the results to similar settings [55]. Additionally, the scope of the technology used in the studies was limited, raising questions about the applicability of findings to other types of voice-activated smart technologies (eg, Google Assistant and Alexa) used in diverse geographical locations and settings [55]. One study noted that subsequent research should aim to include older adults with varying levels of technology expertise and from various demographic backgrounds [55]. For example, Kim and Choudhury [54] noted that the need for future research to focus on addressing voice-activated virtual assistant use among older adults with hearing impairments is crucial to promote inclusivity. This could involve exploring innovative ways to integrate voice-activated functionality discreetly into hearing aids. Exploring how these findings apply to other voice devices is recommended by study authors to broaden the understanding of technology use among older adults [55].

One study noted the challenge of conducting a detailed quantitative analysis of use patterns due to variability in study start dates among participants [54]. Further studies should use a more rigorous recruitment strategy to enhance repeatability and should also endeavor to collect additional information, in a nonintrusive manner, regarding the reasons for participant withdrawal, especially considering that 2 of 6 participants withdrew [53]. This points to the need for methodological approaches that can accommodate and analyze variations in technology use over time.

## Discussion

### Summary of Findings

With an increasing aging population and the rapid expansion of the IoT, there is growing interest in the role of AAL technologies for supporting aging in place [57-59]. This scoping review aimed to investigate the interaction between these technologies and older adults, with an interest in exploring how both technologies and users adapt to enhance user experiences and generate benefits from these technologies over time. Specifically, this study delved into the coadaptation process between older adults and wearables and voice-activated virtual assistants, a critical yet underexplored area in the scientific literature. Despite the importance of this area for informing technology design and intervention development, we found a gap in long-term interaction research, with only 5 studies, mostly from the United States, addressing this topic. These studies focused on the use of voice-activated virtual assistants in the context of commercially available smart speakers, exploring how older adults engage with and adapt to these technologies over short periods of time. We found that older adults often engage in an iterative learning process, adjusting their interaction styles and approaches with technology over time. Participants tend to develop routines for using the technology and adapted their behaviors, such as words to engage with the technology and to navigate challenges encountered during technology use. However, persistent challenges related to communication barriers between older adults and the technologies were observed, impacting user confidence and hindering personalized interactions by the technology. The included studies also highlighted several limitations to existing research, including small sample sizes, limited participant diversity, and constraints associated with the technology used. These limitations suggest the need for further research involving larger and more diverse cohorts of older adults, encompassing various demographics and geographic locations. Future studies should also explore the applicability of findings to different virtual assistants and voice-activated devices.

### Lack of Diversity

Our review identified that within the scarce literature exploring the coadaptation of older adults using voice-activated virtual assistants and wearables, studies tend to reflect a limited diversity of participants across racial, ethnic, cultural, and socioeconomic backgrounds among the participants. The lack of diversity in the context of technology adaptation has been reported in previous reviews [44], and most smart technologies have been trained using native English accents [60]. This lack of diversity in the design process leads to oversights in accommodating various cultural, socioeconomic, linguistic, and individual differences among older adults [61]. Existing studies have noted that often individuals from racialized groups feel that the voices of virtual assistants do not resonate with their culture in terms of tone and dialect [62]. Similarly, technologies often struggle to identify and respond to accents of individuals for whom English is a second (or third) language [63,64]. Scholars have argued that encouraging the active involvement of diverse older adults throughout the design process spanning from conception to product development and deployment is essential to improving smart technologies to meet the needs of diverse older adults [65]. Future research is encouraged to explore how adults of various linguistic, ethnic, cultural, and socioeconomic backgrounds adapt to voice-activated virtual assistants and CAs, especially those without prior experience with these technologies. This future research can help researchers and technology developers better understand the nuanced requirements of older adults. Moreover, our review noted that participants tended to be relatively healthy, with one study including individuals with MCI [52] and another including those with hearing devices, but no communication difficulties [54]. As such, we lack the opportunity to identify the unique needs of older adults with various forms of disability looking to adopt technology to support their aging in place. Our study therefore highlights the necessity of future research to concentrate on evaluating how older adults with various forms of disability and illness coadapt to technology over time. These studies would shed light on the underlying factors contributing to varying adoption rates among older adults, providing insights into the reasons behind the differing adoption speeds of personal health records. Moreover, evaluations of technology use over time should encompass additional demographic indicators, like educational status (only reported in 1 study), to enhance comprehension of the findings among older adult participants. This inclusion would provide a broader context and facilitate better extrapolation of the study outcomes.

### Methods Used for Coadaptation

Our review noted that while papers lacked a precise definition of coevolution or coadaptation, they did note the importance of exploring changes in older adults’ use of smart technologies over time and emphasized the need to further tailor commercially available technologies to meet the specific needs and capacities of older adults. This tailoring will be critical to enhance the realization of anticipated benefits of these technologies for supporting aging in place, considering earlier studies highlight the importance of personalized interactions based on individual preferences and habits [66-68]. However, sustained challenges in prolonged technology use were also identified, primarily stemming from communication barriers between humans and machines, mirroring existing cross-sectional studies [69,70]. Moreover, while the design of commercially available technologies often overlooks elements of communication typical of older adults as noted in other studies [71], the reverse is also true with older adults not always understanding the commands and unique features of these technologies, leading to misunderstandings and frustration. Instructions requiring access to additional platforms or technical knowledge added to the confusion, highlighting the need for clearer, more user-friendly interfaces that incorporate multiple types of affordances to help older adults use smart technologies more easily to accomplish desired tasks, thereby leading to more positive user experiences [72]. Comparatively, future longitudinal research could delve deeper into the nuanced dynamics of human-machine communication and interaction over time (eg, longer than 10 weeks), aiming to bridge the gap between older adults and technology. The absence of a consistent definition of coadaptation across studies suggests the importance of developing a unified framework to facilitate clearer communication and comparison of findings in future research on technology adaptation among older adults.

The included studies in this review revealed how older adults use smart technology changes over time, particularly as they become more familiar with their challenges using technology. Future research in this domain could delve deeper into the bidirectional adaptation process, exploring how AI-driven technological systems can become more responsive, intuitive, and personalized for older adults. This exploration might involve investigating adaptive features, machine learning algorithms, or user interface design tailored explicitly to accommodate the evolving needs and challenges encountered by older adults.

### Limitations

The inclusion criteria of this scoping review required each selected paper to be published in English from 2000 onward, contain primary data, and include mention of coadaptation between an older adult user and technology over time. We used a comprehensive and methodologically rigorous search strategy, developed with expert consultation and peer-reviewed to ensure accuracy and thoroughness. Despite these efforts, the rapidly evolving nature of technology and the broad conceptual scope of coadaptation might limit the absolute inclusivity of our study selection. In particular, the terminology and application in studies involving CAs, smart speakers, and voice-activated virtual assistants are diverse and continuously developing. While we are confident in the relevance and applicability of the studies included, we acknowledge that the field’s dynamic nature may yield additional relevant studies after our review period. This recognition underlines the importance of continuous surveillance and updating of the literature in this domain to capture emerging insights and innovations that could further elucidate the coadaptation processes between older adults and smart technologies. These limitations highlight areas for methodological refinement in future research to ensure broader applicability and understanding of the coadaptation process.

### Conclusions

This scoping review aimed to explore the dynamics of interaction between older adults and 2 prevalent types of smart technologies, wearables, and voice-activated virtual assistants, over time. Through a systematic search across multiple databases using a comprehensive set of keywords, this review endeavored to synthesize existing literature on how older adults engage with these technologies, adapting their use to enhance their daily lives. Despite the rigorous search strategy, the review identified only 5 papers that met the inclusion criteria, revealing a scarcity in the body of literature on this subject.

The findings from the included studies focused on voice-activated virtual assistants and highlighted an iterative learning process undertaken by older adults, who adapt their interaction styles and behaviors to navigate and overcome challenges presented by the technology. Notably, these adaptations reflect a resilience and willingness to integrate technology into their routines, despite encountering persistent communication barriers. These barriers, which often impede personalized interactions and undermine user confidence, highlight critical areas for technological improvement.

Moreover, the review brings to light significant limitations within the existing studies, such as small sample sizes, lack of participant diversity among participants, and a narrow technological focus. These shortcomings signal a pressing need for further research that encompasses broader and more diverse populations of older adults. Such research is essential to developing a deeper understanding of the diverse ways in which older adults from various backgrounds engage with and adapt to smart technologies. By addressing these research gaps, future studies have the potential to significantly contribute to the development of more inclusive, user-friendly, and effective technological solutions tailored to the needs and preferences of the aging population.

In conclusion, this scoping review highlights many unexplored areas for investigation. Expanding the scope of research to include larger, more diverse cohorts and a wider range of technologies will be crucial to advancing the use of smart technology to improve the quality of life and independence of older adults.

## Appendix

Multimedia Appendix 1 PRISMA-ScR (Preferred Reporting Items for Systematic Reviews and Meta-Analyses Extension for Scoping Reviews) checklist.

Multimedia Appendix 2 Search strategies.

Multimedia Appendix 3 Summary of papers excluded and rationale.

## Abbreviations

AI: artificial intelligence
AAL: ambient-assisted living
CA: conversational agent
IoT: Internet of Things
MCI: mild cognitive impairment
PRESS: Peer Review of Electronic Search Strategies
PRISMA: Preferred Reporting Items for Systematic Reviews and Meta-Analyses
PRISMA-ScR: Preferred Reporting Items for Systematic Reviews and Meta-Analyses Extension for Scoping Reviews

## Data Availability

The data sets generated and analyzed during this study are available from the corresponding author upon reasonable request.
