# Supplementary material for: Longitudinal Coadaptation of Older Adults With Wearables and Voice-Activated Virtual Assistants: Scoping Review
Source: J Med Internet Res. 2024 Aug 7;26:e57258. doi: 10.2196/57258 (PMC11339587; doi:10.2196/57258)
Supplement: Multimedia Appendix 2 [file jmir_v26i1e57258_app2.docx]

## OVID Medline:

Ovid MEDLINE(R) ALL <1946 to March 1 2024>

1 Aged/

2 Frail Elderly/

3 "Aged, 80 and over"/

4 Centenarians/

5 Nonagenarians/

6 Octogenarians/

7 Geriatric assessment/

8 ((Old adj2 Adult*) or (Old adj2 Person*) or (Old adj2 People*) or (Old adj2 Patient*) or (Old adj2 Citizen*) or (Older adj2 Adult*) or (Older adj2 Person*) or (Older adj2 People*) or (Older adj2 Patient*) or (Older adj2 Citizen*) or (Oldest adj2 Adult*) or (Oldest adj2 Person*) or (Oldest adj2 People*) or (Oldest adj2 Patient*) or (Oldest adj2 Citizen*) or (Ag?ing adj2 Adult*) or (Ag?ing adj2 Person*) or (Ag?ing adj2 People*) or (Ag?ing adj2 Patient*) or (Ag?ing adj2 Citizen*) or (Aged adj2 Adult*) or (Aged adj2 Person*) or (Aged adj2 People*) or (Aged adj2 Patient*) or (Aged adj2 Citizen*)).tw,kf,kw.

9 (Oldest Old or Elder* or Geriatric* or Senior* or Long-Lived or Over-the-hill or Senescen* or Centenarian or Nonagenarian or Octogenarian or Septuagenarian or Sexagenerian or Unyoung).tw,kf,kw.

10 ((Aged adj "60") or (Aged adj "70") or (Aged adj "75") or (Aged adj "80") or (Aged adj "85") or (Aged adj "90") or (Aged adj "95") or (Aged adj "100") or (Aged adj sixty) or (Aged adj sixty-five) or (Aged adj seventy) or (Aged adj seventy-five) or (Aged adj eighty) or (Aged adj eighty-five) or (Aged adj ninety) or (Aged adj ninety-five) or (Aged adj one hundred) or (Aged adj a hundred)).tw,kf,kw.

11 ((Geriatric adj2 assess*) or (later adj1 life) or (late* adj1 adulthood)).tw,kf,kw.

12 or/1-11

13 Digital Technology/

14 Smartphone/

15 Technology/

16 User-Computer Interface/

17 Wearable Electronic Devices/

18 Voice Recognition/

19 Telemedicine/

20 ((Digital adj2 Technolog*) or (Digital adj2 Health*) or (Digital adj2 Electronic*) or (Digital adj5 intervention*) or (Smart adj2 Phone*)).tw,kf,kw.

21 (Smartphone or technology or technologies or tech).tw,kf,kw.

22 ((user adj3 interface*) or (computer adj3 system*) or (virtual adj2 system*)).tw,kf,kw.

23 ((Wearable* adj3 device*) or (wearable* adj3 electronic*) or (wearable adj2 technolog*) or (wearable* adj3 sensor*) or (wearable* adj3 interface) or (inertia adj1 sensor) or (wireless adj1 sensor) or (inertia adj1 monitor) or (motion adj1 monitor) or (electronic adj1 skin*) or (electronic adj3 device*)).tw,kf,kw.

24 ((Voice adj2 recognition*) or (voice adj2 identification*) or (voice adj2 control*)).tw,kf,kw.

25 (eHealth or e-health or telehealth or telemedicine*).tw,kf,kw.

26 or/13-25

27 (Co-adapt* or coadapt*).mp.

28 (adapt* or personali?ation* or domestication* or integration* or tailoring or configuration or customization or individualization or user-centered or user-centric or user-fitted).tw,kw,kf.

29 ((self-learning adj2 system*) or (selflearning adj2 system*) or (user-oriented adj2 engineering) or (useroriented adj2 engineering) or (user-defined adj2 settings) or (userdefined adj2 settings)).tw,kf,kw.

30 or/27-29

31 12 and 26 and 30
