# Supplementary material for: Longitudinal Coadaptation of Older Adults With Wearables and Voice-Activated Virtual Assistants: Scoping Review
Source: J Med Internet Res. 2024 Aug 7;26:e57258. doi: 10.2196/57258 (PMC11339587; doi:10.2196/57258)
Supplement: Multimedia Appendix 3 [file jmir_v26i1e57258_app3.docx]

| Author, Year | Title | Study does not gather empirical data | Not a voice-activated virtual assistant^[[1]](#footnote-2)^ or wearable technology^[[2]](#footnote-3)^ | No record of older adult adapting to technology | No record of technology adapting to older adult | Time was  not >8 weeks |
| --- | --- | --- | --- | --- | --- | --- |
| Aguilera-Hermida, 2022 | Residents’ perception of the use of smart-home technologies in a retirement community | x | Wearable technology | x | Uses machine learning algorithms to learn from user preferences input via a smartphone app | x |
| Atay et al., 2016 | Can a smartphone-based chatbot engage older community group members? The impact of specialised content | x | x | x | x | x |
| Ballard et al., 2016 | Adapting the Elder Abuse Suspicion Index© for use in the geriatric long-term care setting | Empirical data collected | x | x | x | x |
| Batsis et al., 2017 | Telemedicine and primary care obesity management in rural areas-innovative approach for older adults? | x | x | x | x | x |
| Anuradha et al., 2022 | iFall-An android application for fall monitoring and response | x | x | Users interact with the app through its interface to confirm or dismiss alerts, which could influence their behavior regarding how they carry and interact with their phone | iFall App includes adaptive algorithms for fall detection that adjust based on user input (e.g., sensitivity settings) and potentially on user behavior as it operates (adjusting thresholds based on detected movement patterns). This suggests adaptation where the app adjusts to better suit the detection of falls based on how the phone is carried or used | x |
| Beristain et al., 2022 | User centered virtual coaching for older adults at home using SMART goal plans and I-Change model | x | Voice-activated virtual assistant | Users adapt by setting and personalizing SMART goals, participating in the interventions, and providing feedback which the system then incorporates. This indicates behavioral changes influenced by the system | Enhanced machine learning algorithm that supports momentary intervention planning, adapting interventions based on user feedback and behaviors in real-time | x |
| Bhattarai et al., 2017 | The role of digital health technologies in management of pain in older people: An integrative review. | x | x | x | x | x |
| Borelli et al., 2019 | HABITAT: An IoT solution for independent elderly sensors | x | Wearable technology | Includes AI modules that process data from smart devices to adapt responses and interactions based on user behavior | x | x |
| Burns et al., 2016 | PESTO: Data integration for visualization and device control in the SmartCare project | x | x | x | x | x |
| Cabrita et al., 2019 | Older adults' attitudes toward ambulatory technology to support monitoring and coaching of healthy behaviors: Qualitative study | Empirical data collected | Wearable technology | x | x | x |
| Cheng et al., 2022 | Canadian integrated health and social care case studies: Success factors that support integration | Empirical data collected | x | x | x | x |
| Choi et al., 2020 | Internet and health information technology use and psychological distress among older adults with self-reported vision impairment: Case control study | Empirical data collected | x | Evidence that users (older adults with visual impairment) adapted their behaviors in response to technology, primarily through their engagement with health information technology and its potential influence on their psychological distress levels | x | x |
| Claes et al., 2015 | Attitudes and perceptions of adults of 60 years and older towards in-home monitoring of the activities of daily living with contactless sensors: An explorative study | x | x | x | x | x |
| Cochrane et al., 2016 | Time-limited homecare reablement services for maintaining and improving the functional independence of older adult | Empirical data collected | x | x | x | x |
| Edlin-White et al., 2011 | Accessibility for older users through adaptive interfaces: Opportunities, challenges, and achievements | Empirical data collected | x | x | Designed to dynamically adjust the user interface based on sensed user behaviors and inferred need | x |
| Fayed et al., 2023 | Immersion and presence in virtual reality applications for physical therapy and upper limb rehabilitation | x | x | x | x | x |
| Gagnon-Roy et al., 2017 | Assistive technology addressing safety issues in dementia: A scoping review | x | x | x | x | x |
| Guimarães et al., 2021 | An exergame solution for personalized multicomponent training in older adults | x | Wearable technology | Designed to automatically adapt the difficulty and load of exercises based on user performance and progression | Users adapt their behavior to the requirements of the exergame, potentially leading to improvements in physical and cognitive functions | x |
| Heur et al., 2011 | Active error corrections enhance adaptation to a visuo-motor rotation | Empirical data collected | x | Participants adapt their motor responses based on the feedback and resistance provided by the robot | Robots adjusting the force and direction based on the participant's movements | x |
| Holthe et al., 2020 | The assisted living project: A process evaluation of implementation of sensor technology in community assisted living. A feasibility study | Empirical data collected | x | x | x | x |
| Hsiao et al., 2017 | User interface based on natural interaction design for seniors | Empirical data collected | x | x | Adapts to the users through gesture recognition, suggests a form of adaptation where the system responds to user behavior | x |
| Infarinato et al., 2020 | Acceptance and potential impact of the e-wall platform for health monitoring and promotion in persons with a chronic disease or age-related impairment | Empirical data collected | x | Participants adapted their behavior in response to the feedback and suggestions provided by the eWALL platform | eWALL system adapts by providing personalized feedback | x |
| Johnston et al., 2009 | Designing and testing a web-based interface for self-monitoring of exercise and symptoms for older adults with chronic obstructive pulmonary disease | Empirical data collected | x | Interaction is intended to encourage consistent self-management practices, including tracking and responding to symptoms | x | x |
| Kaldenberg and Smallfield, 2020 | Occupational therapy practice guidelines for older adults with low vision | Empirical data collected | x | x | x | 8 weeks |
| Kamin et al., 2017 | Subjective technology adaptivity predicts technology use in old age | Empirical data collected | x | x | x | x |
| Klemets et al., 2019 | Integration of an in-home monitoring system into home care nurses’ workflow: A case study | Empirical data collected | x | Nurses integrate the technology into their daily workflows, gradually seeing its benefits and relying on its data | Uses passive infrared sensors installed in various rooms of an apartment to monitor the movements of elderly clients, particularly during the night | x |
| Kliesch et al., 2023 | Evaluation of two self-fitting user interfaces for bimodal CI-recipients | Empirical data collected | x | x | x | x |
| Koch et al., 2014 | Informatics and socio-technical challenges when designing solutions for integrated eCare | x | x | x | x | x |
| Kucharski et al., 2022 | Relationships between ICT use and subjective well-being among the oldest-old in Germany: Findings from the NRW80+study | Empirical data collected | Voice activated virtual assistant | x | x | 4 months |
| Lee et al., 2020 | Adapting “Sunshine” a socially assistive chat robot for older adults with cognitive impairment: A pilot study | Empirical data collected | x | Adapting their social behaviors and potentially enhancing their quality of life through interactive play and conversation | Adaptation seems primarily driven by pre-programmed responses rather than dynamic, algorithm-driven changes adapting in real-time to the user's changing behaviors and preferences | Up to 3 months |
| Loveys et al., 2021 | A digital human for delivering a remote loneliness and stress intervention to at-risk younger and older adults during the COVID-19 pandemic: Randomized pilot trial | Empirical data collected | Voice activated virtual assistant | x | Uses computer-generated imaging and artificial intelligence designed to interact in a personalized manner | x |
| Lowrey et al., 2022 | Impairments in cognitive control using a reverse visually guided reaching task following stroke | Empirical data collected | x | x | x | x |
| Lunardini et al., 2017 | Exergaming for balance training, transparent monitoring, and social inclusion of community-dwelling elderly | x | x | User expected to engage using the virtual community for social interaction and participating in tailored cognitive and physical activities | x | Up to 4 months |
| Mannheim et al., 2023 | Ageism in the discourse and practice of designing digital technology for older persons: A scoping review | x | x | x | x | x |
| Marin et al., 2014 | Social networking sites photos and robots: A pilot research on Facebook photo albums and robotics interfaces for older adults | Empirical data collected | Voice activated virtual assistant | x | x | x |
| Menghi et al., 2018 | Assessment of a smart kitchen to help people with Alzheimer’s disease | Empirical data collected | x | x | x | x |
| Menghi et al., 2019 | Product service platform to improve care systems for elderly living at home | Empirical data collected | x | x | x | x |
| Mitchell et al., 2020 | “It’s like a cyber-security blanket”: The utility of remote activity monitoring in family dementia care | Empirical data collected | x | x | x | Up to 18 months |
| Naccarelli et al., 2023 | e-VITA use cases configurator: A tool to identify the optimal configuration of the sensor network and coaching devices to enable older people to age well at home | x | Voice activated virtual assistant and wearable sensors | x | The e-VITA virtual coach offers personalized recommendations based on the analysis of data collected from wearable devices and sensors placed in the smart living environment and provides support through natural language interaction with holograms, emotional objects, or robots | x |
| Naccarelli et al., 2022 | Using a smart living environment simulation tool and machine learning to optimize the home sensor network configuration for measuring the activities of daily living of older people | Empirical data collected | x | x | x | x |
| Naick et al., 2017 | Innovative approaches of using assistive technology to support carers to care for people with night-time incontinence issues | Empirical data collected | x | x | x | x |
| Nakamura et al., 2022 | Digital psychosocial intervention for depression among older adults in socioeconomically deprived areas in Brazil (PRODIGITAL-D): Protocol for an individually randomised controlled trial | x | x | x | x | x |
| National Opinion Research Center | Evaluating the cost effectiveness of STEADI | x | x | x | x | 12 months |
| Ng et al., 2017 | A qualitative case study of smartphone-connected hearing aids: Influences on patients, clinicians, and patient–clinician interactions | Empirical data collected | x | x | x | x |
| Nota et al., 2007 | Self-determination, social abilities, and the quality of life of people with intellectual disability | Empirical data collected | x | x | x | x |
| Nyandara., 2012 | Challenges and opportunities of technology-based instruction in open and distance learning: a comparative study of Tanzania and China | Empirical data collected | x | x | x | x |
| O’Brien et al., 2022 | Optimizing voice‐controlled intelligent personal assistants for use by home‐bound older adults | Empirical data collected | Voice activated virtual assistant | x | x | x |
| Odeh et al., 2014 | Acceptability of telehealth by elderly patients | Empirical data collected | x | x | x | x |
| Palmer et al., 2006 | Evaluation of a second-order directional microphone hearing aid: II. self-report outcomes | Empirical data collected | Wearable technology | x | x | x |
| Park et al., 2022 | The impact of everyday AI-based smart speaker use on the well-being of older adults living alone | Empirical data collected | Voice activated virtual assistant | x | Adaptation involves integrating the speaker into their daily routines and relying on it for social interaction and emotional support, which seems to influence their levels of depression and loneliness positively | 8 weeks |
| Pereira et al., 2020 | The diffusion of gerontechnology for fall prevention, fall detection, and fall monitoring model testing | Empirical data collected | x | x | x | x |
| Pérez-Rodríguez et al., 2021 | Usability, user experience, and acceptance evaluation of CAPACITY: A technological ecosystem for remote follow-up of frailty | Empirical data collected | x | x | x | x |
| Portz et al., 2020 | Using grounded theory to inform the human-centered design of digital health in geriatric palliative care | Empirical data collected | x | x | x | x |
| Pradhan et al., 2019 | “Phantom friend or just a box with information" personification and ontological categorization of smart speaker-based voice assistants by older adults | Empirical data collected | Voice activated virtual assistant | x | x | x |
| Procter et al., 2014 | The day-to-day co-production of ageing in place. Computer Supported Cooperative Work | Empirical data collected | x | x | x | x |
| Pulik et al., 2022 | First Polish mobile application for patients undergoing total hip arthroplasty | x | x |  | x | x |
| Rábago et al., 2011 | Application of a mild traumatic brain injury rehabilitation program in a virtual realty environment: A case study | Empirical data collected | x | x | x | x |
| Radhakrishnan et al., 2020 | Personalizing sensor-controlled digital gaming to self-management needs of older adults with heart failure: A qualitative study | Empirical data collected | x | x | x | x |
| Rath et al., 2021 | Leveraging voice assistive technology to enhance health monitoring of older adults | Empirical data collected | Voice activated virtual assistant and wearable technology | x | x | x |
| Rejeski et al., 2013 | Promoting physical activity for elders with compromised function: the lifestyle interventions and independence for elders (LIFE) study physical activity intervention | Empirical data collected | x | x | x | x |
| Requena-Komuro et al., 2022 | Remote versus face-to-face neuropsychological testing for dementia research: A comparative study in people with Alzheimer’s disease, frontotemporal dementia and healthy older individuals | Empirical data collected | x | x | x | x |
| Rodríguez et al., 2009 | Home-based communication system for older adults and their remote family | Empirical data collected | x | x | x | x |
| Rutgers University | Use surveillance technology to reduce elder abuse recidivism | x | x | x | x | x |
| Sala et al., 2018 | Video game training does not enhance cognitive ability: A comprehensive meta-analytic investigation | Empirical data collected | x | x | x | x |
| Sancar et al., 2016 | Towards ability-based optimization for aging users | x | x | x | x | x |
| Sarcar et al., 2020 | Evaluation of model-based optimized touchscreen keyboard for older adults with hand tremor. | Empirical data collected | x | x | x | x |
| Scanlon et al., 2020 | Stylistic variation in African American language: Examining the social meaning of linguistic features in a Seattle community | Empirical data collected | x | x | x | x |
| Scherr et al., 2020 | Alexa, tell me more-about new best friends, the advantage of hands-free operation and life-long learning | Empirical data collected | Voice activated virtual assistant | x | x | 12 months |
| Schlomann et al., 2017 | A case study on older adults' long-term use of an activity tracker | Empirical data collected | Wearable sensor | Participants had adjusted daily habits, behaviour, or physical activity goals according to the feedback of the activity tracker | x | 12 months |
| Shahal et al., 2022 | User-specific touch interfaces: A viable solution for an aging society? | Empirical data collected | x | x | x | x |
| Sheppard et al., 2023 | Access to Community Support Services among Older Adults in Social Housing in Ontario | Empirical data collected | x | x | x | x |
| Sifan et al., 2021 | Design and development of intelligent assistant mobile application for the elderly | Empirical data collected | x | x | x | x |
| Stahl, 2019 | Digital monitoring of sleep, meals, and physical activity as a preventive intervention for depression in older bereaved adults: A pilot study on feasibility, acceptability, and symptom | x | x | Improved quality of life measures | x | 12 or more weeks |
| Stauder, 2021 | Telemedicine in the care of geriatric oncological patients | x | x | x | x | x |
| Stawarz et al., 2023 | Exploring the potential of technology to promote exercise snacking for older adults who are prefrail in the home setting: User-centered design study | Empirical data collected | x | x | x | x |
| Summers et al., 2018 | The My Active and Healthy Aging (My-AHA) ICT platform to detect and prevent frailty in older adults: Randomized control trial design and protocol | x | x | x | x | 18 months |
| Thomas et al., 2014 | An older adult perspective on digital legacy | x | x | x | x | x |
| Tinker et al., 2005 | Introducing assistive technology into the existing homes of older people: Feasibility, acceptability, costs, and outcomes | x | x | Improved independence | x | x |
| Tinker et al., 2004 | Introducing assistive technology (AT) into the homes of older people: The REKI (REading KIng’s) research project | Empirical data collected | x | x | x | x |
| Tsai et al., 2019 | Senior technology exploration, learning, and acceptance (STELA) model: From exploration to use-a longitudinal randomized controlled trial | Empirical data collected | x | Increased usage | x | 2 months |
| van Leersum et al., 2023 | Engaging older adults with a migration background to explore the usage of digital technologies in coping with dementia | Empirical data collected | x | x | x | 4 months |
| Velazquez et al., 2013 | Design of exergames with the collaborative participation of older adults | Empirical data collected | x | x | x | 2 months |
| Warner et al., 2022 | Everyday assistive technologies provide meaningful support to persons with dementia and their informal caregivers? Evaluation of collaborative community program | Empirical data collected | x | Improved independence | x | x |
| Williams et al., 2016 | Adapting telemonitoring technology use for older adults: A pilot Study | Empirical data collected | x | x | x | x |
| Williams et al., 2018 | Senior citizens usage towards and perception of modern technology in India | x | x | x | x | x |
| Wu & Hu, 2018 | A study on the behavior of using intelligent television among the elderly in new urban areas | Empirical data collected | x | x | x | x |
| Yamazaki et al., 2023 | Long-term effect of the absence of a companion robot on older adults: A preliminary pilot study | Empirical data collected | x | Participants reported psychological distress when the robot was removed, indicating adaptation to its companionship | x | Yes, for 11/13 patients |
| Yücel et al., 2022 | I am old too!: Understanding the impact of empathy and voice characteristics on older adults' perception of voice assistants | Empirical data collected | Voice-activated virtual assistant | Adaptation in communication style | x | x |
| Zheng et al., 2018 | A web-based treatment decision support tool for patients with advanced knee arthritis: Evaluation of interface and content design | Empirical data collected | x | x | x | x |
| Zhou et al., 2023 | How to increase consumers’ continued use intention of artificial intelligence voice assistants? The role of anthropomorphic features | Empirical data collected | Voice-activated virtual assistant | x | x | x |
| Zhu et al., 2022 | Learning and daily life integration: A qualitative analysis of the behaviors, characteristics, and logic of mobile learning among older adults | Empirical data collected | x | x | x | x |

1. Aguilera-Hermida AP. Residents' perception of the use of smart-home technologies in a retirement community. Gerontechnology. 2022 Oct 1;21.
2. Aldaz G. *Smartphone-Based System for Learning and Inferring Hearing Aid Settings*. ProQuest Dissertations Publishing; 2016.
3. Atay C, Ireland D, Liddle J, et al. CAN A SMARTPHONE-BASED CHATBOT ENGAGE OLDER COMMUNITY GROUP MEMBERS? THE IMPACT OF SPECIALISED CONTENT. *Alzheimer’s & dementia*. 2016;12(7):P1005-P1006. doi:10.1016/j.jalz.2016.06.2070
4. Ballard S. *Adapting the Elder Abuse Suspicion Index© for Use in the Geriatric Long-Term Care Setting*. ProQuest Dissertations Publishing; 2016.
5. Batsis JA, Pletcher SN, Stahl JE. Telemedicine and primary care obesity management in rural areas - innovative approach for older adults? *BMC geriatrics*. 2017;17(1):6-6. doi:10.1186/s12877-016-0396-x
6. Beristain Iraola A, Álvarez Sánchez R, Hors-Fraile S, et al. Correction: Beristain Iraola et al. User Centered Virtual Coaching for Older Adults at Home Using SMART Goal Plans and I-Change Model. Int. J. Environ. Res. Public Health 2021, 18 , 6868. *International journal of environmental research and public health*. 2022;19(4):2116-. doi:10.3390/ijerph19042116
7. Bhattarai P, Phillips JL. The role of digital health technologies in management of pain in older people: An integrative review. *Archives of gerontology and geriatrics*. 2017;68:14-24. doi:10.1016/j.archger.2016.08.008
8. Borelli E, Paolini G, Antoniazzi F, et al. HABITAT: An IoT Solution for Independent Elderly. *Sensors (Basel, Switzerland)*. 2019;19(5):1258-. doi:10.3390/s19051258
9. Burns NB, Sassaman P, Daniel K, Huber M, Zaruba G. PESTO: Data integration for visualization and device control in the SmartCare project. In: *2016 IEEE International Conference on Pervasive Computing and Communication Workshops (PerCom Workshops)*. IEEE; 2016:1-6. doi:10.1109/PERCOMW.2016.7457137
10. Cabrita M, Tabak M, Vollenbroek-Hutten MM. Older Adults’ Attitudes Toward Ambulatory Technology to Support Monitoring and Coaching of Healthy Behaviors: Qualitative Study. *JMIR aging*. 2019;2(1):e10476-e10476. doi:10.2196/10476
11. Cheng SM. Canadian Integrated Health and Social Care Case Studies: success factors that support integration. International Journal of Integrated Care (IJIC). 2022 Apr 2;22.
12. Choi NG, DiNitto DM, Lee OE, Choi BY. Internet and Health Information Technology Use and Psychological Distress Among Older Adults With Self-Reported Vision Impairment: Case-Control Study. *Journal of medical Internet research*. 2020;22(6):e17294-e17294. doi:10.2196/17294
13. Claes V, Devriendt E, Tournoy J, Milisen K. Attitudes and perceptions of adults of 60 years and older towards in-home monitoring of the activities of daily living with contactless sensors: An explorative study. *International journal of nursing studies*. 2015;52(1):134-148. doi:10.1016/j.ijnurstu.2014.05.010
14. Cochrane A, Furlong M, McGilloway S, Molloy DW, Stevenson M, Donnelly M. Time-limited home-care reablement services for maintaining and improving the functional independence of older adults. *Cochrane database of systematic reviews*. 2016;10(10):CD010825-CD010825. doi:10.1002/14651858.CD010825.pub2
15. Dr. Anuradha SG, Tanuja S, Sadiqa Noorani, M. Sangeetha. iFall-An Android Application for Fall Monitoring and Response. *International Journal of Advanced Research in Science, Communication and Technology*. Published online 2022:161-164. doi:10.48175/IJARSCT-5317
16. Edlin-White R, Cobb S, D’Cruz M, Floyde A, Lewthwaite S, Riedel J. Accessibility for Older Users through Adaptive Interfaces: Opportunities, Challenges and Achievements. In: *Human-Computer Interaction. Towards Mobile and Intelligent Interaction Environments*. Springer Berlin Heidelberg; :483-489. doi:10.1007/978-3-642-21616-9_54
17. Fayed M, Almadi F, Almadi M, et al. Immersion and Presence in Virtual Reality Applications for Physical Therapy and Upper Limb Rehabilitation. In: *Social Computing and Social Media*. Springer Nature Switzerland; :217-227. doi:10.1007/978-3-031-35927-9_16
18. Gagnon-Roy M, Bourget A, Stocco S, Courchesne ACL, Kuhne N, Provencher V. Assistive Technology Addressing Safety Issues in Dementia: A Scoping Review. *The American journal of occupational therapy*. 2017;71(5):7105190020p1-7105190020p10. doi:10.5014/ajot.2017.025817
19. Guimarães V, Oliveira E, Carvalho A, et al. An Exergame Solution for Personalized Multicomponent Training in Older Adults. *Applied sciences*. 2021;11(17):7986-. doi:10.3390/app11177986
20. Gvozdanovic A, Jozsa F, Fersht N, et al. Integration of a personalised mobile health (mHealth) application into the care of patients with brain tumours: proof-of-concept study (IDEAL stage 1). *BMJ surgery, interventions, & health technologies*. 2022;4(1):e000130-e000130. doi:10.1136/bmjsit-2021-000130
21. Heuer H, Rapp K. Active error corrections enhance adaptation to a visuo-motor rotation. *Experimental brain research*. 2011;211(1):97-108. doi:10.1007/s00221-011-2656-5
22. Holthe T, Casagrande FD, Halvorsrud L, Lund A. The assisted living project: a process evaluation of implementation of sensor technology in community assisted living. A feasibility study. *Disability and rehabilitation: Assistive technology*. 2020;15(1):29-36. doi:10.1080/17483107.2018.1513572
23. Hsiao SW, Lee CH, Yang MH, Chen RQ. User interface based on natural interaction design for seniors. *Computers in human behavior*. 2017;75:147-159. doi:10.1016/j.chb.2017.05.011
24. Infarinato F, Jansen-Kosterink S, Romano P, et al. Acceptance and Potential Impact of the eWALL Platform for Health Monitoring and Promotion in Persons with a Chronic Disease or Age-Related Impairment. *International journal of environmental research and public health*. 2020;17(21):7893-. doi:10.3390/ijerph17217893
25. Johnston SK, Nguyen HQ, Wolpin S. Designing and Testing a Web-based Interface for Self-Monitoring of Exercise and Symptoms for Older Adults with COPD. *Computers, informatics, nursing*. 2009;27(3):166-174. doi:10.1097/NCN.0b013e31819f7c1d
26. Kaldenberg J, Smallfield S. Occupational Therapy Practice Guidelines for Older Adults With Low Vision. *The American journal of occupational therapy*. 2020;74(2):7402397010p1-7402397010p23. doi:10.5014/ajot.2020.742003
27. Kamin ST, Lang FR, Beyer A. Subjective Technology Adaptivity Predicts Technology Use in Old Age. *Gerontology (Basel)*. 2017;63(4):385-392. doi:10.1159/000471802
28. Kim D. Can healthcare apps and smart speakers improve the health behavior and depression of older adults? A quasi-experimental study. *Frontiers in digital health*. 2023;5:1117280-1117280. doi:10.3389/fdgth.2023.1117280
29. Klemets J, Määttälä J, Hakala I. Integration of an in-home monitoring system into home care nurses’ workflow: A case study. *International journal of medical informatics (Shannon, Ireland)*. 2019;123:29-36. doi:10.1016/j.ijmedinf.2018.12.006
30. Kliesch S, Chalupper J, Lenarz T, Büchner A. Evaluation of Two Self-Fitting User Interfaces for Bimodal CI-Recipients. *Applied sciences*. 2023;13(14):8411-. doi:10.3390/app13148411
31. Lee OE, Davis B. Adapting “Sunshine,” A Socially Assistive Chat Robot for Older Adults with Cognitive Impairment: A Pilot Study. *Journal of gerontological social work*. 2020;63(6-7):696-698. doi:10.1080/01634372.2020.1789256
32. Loveys K, Sagar M, Pickering I, Broadbent E. A Digital Human for Delivering a Remote Loneliness and Stress Intervention to At-Risk Younger and Older Adults During the COVID-19 Pandemic: Randomized Pilot Trial. *JMIR mental health*. 2021;8(11):e31586-e31586. doi:10.2196/31586
33. Lowrey CR, Dukelow SP, Bagg SD, Ritsma B, Scott SH. Impairments in Cognitive Control Using a Reverse Visually Guided Reaching Task Following Stroke. *Neurorehabilitation and neural repair*. 2022;36(7):449-460. doi:10.1177/15459683221100510
34. Lunardini F, Basilico N, Ambrosini E, Essenziale J, Mainetti R, Pedrocchi A, Daniele K, Marcucci M, Mari D, Ferrante S, Borghese NA. Exergaming for balance training, transparent monitoring, and social inclusion of community-dwelling elderly. In2017 IEEE 3rd International Forum on Research and Technologies for Society and Industry (RTSI) 2017 Sep 11 (pp. 1-5). IEEE.
35. Mannheim I, Wouters EJM, Köttl H, van Boekel LC, Brankaert R, van Zaalen Y. Ageism in the Discourse and Practice of Designing Digital Technology for Older Persons: A Scoping Review. *The Gerontologist*. 2023;63(7):1188-1200. doi:10.1093/geront/gnac144
36. Marin Mejia A. Social networking sites photos and robots: a pilot research on facebook photo albums and robotics interfaces for older adults. In: *Proceedings of the Second International Conference on Human-Agent Interaction*. ACM; 2014:287-291. doi:10.1145/2658861.2658944
37. Menghi R, Gullà F, Germani M. Assessment of a Smart Kitchen to Help People with Alzheimer’s Disease. In: *Smart Homes and Health Telematics, Designing a Better Future: Urban Assisted Living*. Springer International Publishing; :304-309. doi:10.1007/978-3-319-94523-1_30
38. Menghi R, Papetti A, Germani M. Product Service Platform to improve care systems for elderly living at home. *Health policy and technology*. 2019;8(4):393-401. doi:10.1016/j.hlpt.2019.10.004
39. Mitchell LL, Peterson CM, Rud SR, et al. “It’s Like a Cyber-Security Blanket”: The Utility of Remote Activity Monitoring in Family Dementia Care. *Journal of applied gerontology*. 2020;39(1):86-98. doi:10.1177/0733464818760238
40. Naccarelli R, Casaccia S, Homma K, Bevilacqua R, Revel GM. e-VITA Use Cases Configurator: A Tool to Identify the Optimal Configuration of the Sensor Network and Coaching Devices to Enable Older People to Age Well at Home. In: *2023 IEEE International Workshop on Metrology for Living Environment (MetroLivEnv)*. IEEE; 2023:196-201. doi:10.1109/MetroLivEnv56897.2023.10164067
41. Naccarelli R, Casaccia S, Pirozzi M, Revel GM. Using a Smart Living Environment Simulation Tool and Machine Learning to Optimize the Home Sensor Network Configuration for Measuring the Activities of Daily Living of Older People. *Buildings (Basel)*. 2022;12(12):2213-. doi:10.3390/buildings12122213
42. Naick M. Innovative approaches of using assistive technology to support carers to care for people with night-time incontinence issues. World Federation of Occupational Therapists Bulletin. 2017 Jul 3;73(2):128-30.
43. Nakamura CA, Scazufca M, Moretti FA, et al. Digital psychosocial intervention for depression among older adults in socioeconomically deprived areas in Brazil (PRODIGITAL-D): protocol for an individually randomised controlled trial. *Trials*. 2022;23(1):761-761. doi:10.1186/s13063-022-06623-z
44. National Opinion Research Center. Evaluating the Cost Effectiveness of STEADI (STEADI). ClinicalTrials.gov Identifier: NCT05390736. Available from: https://clinicaltrials.gov/ct2/show/NCT05390736. Accessed November 9, 2024
45. Ng SL, Phelan S, Leonard M, Galster J. A Qualitative Case Study of Smartphone-Connected Hearing Aids: Influences on Patients, Clinicians, and Patient–Clinician Interactions. *Journal of the American Academy of Audiology*. 2017;28(6):506-521. doi:10.3766/jaaa.15153
46. Nota L, Ferrari L, Soresi S, Wehmeyer M. Self-determination, social abilities and the quality of life of people with intellectual disability. *Journal of intellectual disability research*. 2007;51(11):850-865. doi:10.1111/j.1365-2788.2006.00939.x
47. Nyandara ZI. Challenges and opportunities of technology-based instruction in open and distance learning: a comparative study of Tanzania and China. In: Proceedings and Report of the 5th UbuntuNet Alliance Annual Conference. 2012:130-145. ISSN: 2223-7062.
48. O’Brien K, Light SW, Bradley S, Lindquist L. Optimizing voice‐controlled intelligent personal assistants for use by home‐bound older adults. *Journal of the American Geriatrics Society (JAGS)*. 2022;70(5):1504-1509. doi:10.1111/jgs.17625
49. Odeh B, Philips N, Kayyali R, Elnabhani S, Griffiths C, Wigmore B, Robinson P, Wallace C. 4 ACCEPTABILITY OF TELEHEALTH BY ELDERLY PATIENTS. Age & Ageing. 2014 Jun 2;43.
50. Palmer C, Bentler R, Mueller GH. Evaluation of a Second-Order Directional Microphone Hearing Aid: II. Self-Report Outcomes. *Journal of the American Academy of Audiology*. 2006;17(3):190-201. doi:10.3766/jaaa.17.3.5
51. Park S, Kim B. The impact of everyday AI-based smart speaker use on the well-being of older adults living alone. *Technology in society*. 2022;71:102133-. doi:10.1016/j.techsoc.2022.102133
52. Pereira GF, Kang M. The diffusion of gerontechnology for fall prevention, fall detection, and fall monitoring model testing. Gerontechnology. 2020 Oct 2;19.
53. Pérez-Rodríguez R, Villalba-Mora E, Valdés-Aragonés M, et al. Usability, User Experience, and Acceptance Evaluation of CAPACITY: A Technological Ecosystem for Remote Follow-Up of Frailty. *Sensors (Basel, Switzerland)*. 2021;21(19):6458-. doi:10.3390/s21196458
54. Portz JD, Ford KL, Doyon K, et al. Using Grounded Theory to Inform the Human-Centered Design of Digital Health in Geriatric Palliative Care. *Journal of pain and symptom management*. 2020;60(6):1181-1192.e1. doi:10.1016/j.jpainsymman.2020.06.027
55. Pradhan A, Findlater L, Lazar A. " Phantom Friend" or" Just a Box with Information" Personification and Ontological Categorization of Smart Speaker-based Voice Assistants by Older Adults. Proceedings of the ACM on human-computer interaction. 2019 Nov 7;3(CSCW):1-21.
56. Procter R, Greenhalgh T, Wherton J, Sugarhood P, Rouncefield M, Hinder S. The day-to-day co-production of ageing in place. Computer Supported Cooperative Work (CSCW). 2014 Jun;23(3):245-67.
57. Pulik Ł, Romaniuk K, Dyrek N, Grabowska N, Łęgosz P. First Polish mobile application for patients undergoing total hip arthroplasty. *Reumatologia*. 2022;60(3):224-228. doi:10.5114/reum.2022.117844
58. Rábago CA, Wilken JM. Application of a Mild Traumatic Brain Injury Rehabilitation Program in a Virtual Realty Environment: A Case Study. *Journal of neurologic physical therapy*. 2011;35(4):185-193. doi:10.1097/NPT.0b013e318235d7e6
59. Radhakrishnan K, Baranowski T, O'Hair M, Fournier CA, Spranger CB, Kim MT. Personalizing sensor-controlled digital gaming to self-management needs of older adults with heart failure: a qualitative study. Games for Health Journal. 2020 Aug 1;9(4):304-10.
60. Rath S, Chandna S. Leveraging voice assistive technology to enhance health monitoring of older adults. In: Proceedings of the 2021 International Conference on Health Informatics. 2021. ISBN: 978-989-8704-31-3.
61. Rejeski WJ, Axtell R, Fielding R, Katula J, King AC, Manini TM, Marsh AP, Pahor M, Rego A, Tudor-Locke C, Newman M. Promoting physical activity for elders with compromised function: the lifestyle interventions and independence for elders (LIFE) study physical activity intervention. Clinical interventions in aging. 2013 Sep 12:1119-31.
62. Requena-Komuro MC, Jiang J, Dobson L, et al. Remote versus face-to-face neuropsychological testing for dementia research: a comparative study in people with Alzheimer’s disease, frontotemporal dementia and healthy older individuals. *BMJ open*. 2022;12(11):e064576-e064576. doi:10.1136/bmjopen-2022-064576
63. Rodríguez MD, Gonzalez VM, Favela J, Santana PC. Home-based communication system for older adults and their remote family. *Computers in human behavior*. 2009;25(3):609-618. doi:10.1016/j.chb.2008.08.017
64. Rutgers University. Use Surveillance Technology to Reduce Elder Abuse Recidivism (MC). ClinicalTrials.gov Identifier: NCT04563728. Available from: https://clinicaltrials.gov/ct2/show/NCT04563728. Accessed February 15, 2024.
65. Sala G, Tatlidil KS, Gobet F. Video Game Training Does Not Enhance Cognitive Ability: A Comprehensive Meta-Analytic Investigation. *Psychological bulletin*. 2018;144(2):111-139. doi:10.1037/bul0000139
66. Sarcar S, Jokinen J, Oulasvirta A, Silpasuwanchai C, Wang Z, Ren X. Towards ability-based optimization for aging users. In: Proceedings of the International Symposium on Interactive Technology and Ageing Populations. 2016:77-86.
67. Sarcar S. Evaluation of model-based optimized touchscreen keyboard for older adults with hand tremor. In: Proceedings of the 6th International ACM In-Cooperation HCI and UX Conference. 2020:11-15.
68. Scandurra I, Koch S, Hägglund M. Informatics and Socio-Technical Challenges when Designing Solutions for Integrated eCare. In: *Healthcare Ethics and Training : Concepts, Methodologies, Tools, and Applications*. ; 2017:261-289. doi:10.4018/978-1-5225-2237-9.ch011
69. Scanlon M. *Stylistic Variation in African American Language: Examining the Social Meaning of Linguistic Features in a Seattle Community*. ProQuest Dissertations Publishing; 2020.
70. Scherr SA, Meier A, Cihan S. Alexa, tell me more–about new best friends, the advantage of hands-free operation and life-long learning. <https://dl.gi.de/server/api/core/bitstreams/526ca45d-f7c5-41f8-ae3b-277fde2b38e3/content>
71. Schlomann A, Seifert A, Zank S, Woopen C, Rietz C. Relationships between ICT use and subjective well-being among the oldest-old in Germany: Findings from the NRW80+ study. Gerontechnology. 2020 Oct 2;19.
72. Schlomann A. A case study on older adults' long-term use of an activity tracker. Gerontechnology. 2017 Mar 1;16(2).
73. Shahal A, Spang RP, Minge M, Trahms C, Voigt-Antons JN. User-specific touch interfaces: a viable solution for an aging society? *Behaviour & information technology*. 2022;41(9):1928-1940. doi:10.1080/0144929X.2021.1906322
74. Sheppard CL, Yau M, Semple C, et al. Access to Community Support Services among Older Adults in Social Housing in Ontario. *Canadian journal on aging*. 2023;42(2):217-229. doi:10.1017/S0714980822000332
75. Sifan W, Kengda H, Xuanyu L, Gang C. Design and Development of Intelligent Assistant Mobile Application for the Elderly. In2021 16th International Conference on Computer Science & Education (ICCSE) 2021 Aug 17 (pp. 543-547). IEEE.
76. Stahl S. DIGITAL MONITORING OF SLEEP, MEALS, AND PHYSICAL ACTIVITY AS A PREVENTIVE INTERVENTION FOR DEPRESSION IN OLDER BEREAVED ADULTS: A PILOT STUDY ON FEASIBILITY, ACCEPTABILITY, AND SYMPTOM IMPROVEMENT. The American Journal of Geriatric Psychiatry. 2019 Mar 1;27(3):S155.
77. Stauder R. Telemedicine in the care of geriatric oncological patients. Oncology Research and Treatment. 2021:28-9.
78. Stawarz K, Liang IJ, Alexander L, Carlin A, Wijekoon A, Western MJ. Exploring the Potential of Technology to Promote Exercise Snacking for Older Adults Who Are Prefrail in the Home Setting: User-Centered Design Study. *JMIR aging*. 2023;6:e41810-e41810. doi:10.2196/41810
79. Summers MJ, Rainero I, Vercelli AE, et al. The My Active and Healthy Aging (My-AHA) ICT platform to detect and prevent frailty in older adults: Randomized control trial design and protocol. *Alzheimer’s & dementia : translational research & clinical interventions*. 2018;4(1):252-262. doi:10.1016/j.trci.2018.06.004
80. Thomas L, Briggs P. An older adult perspective on digital legacy. In: Proceedings of the 8th Nordic Conference on Human-Computer Interaction: Fun, Fast, Foundational. 2014:237-246.
81. Tinker A, Lansley P. Introducing assistive technology into the existing homes of older people: Feasibility, acceptability, costs and outcomes. *Journal of telemedicine and telecare*. 2005;11(1_suppl):1-3. doi:10.1258/1357633054461787
82. Tinker A. Introducing assistive technology (AT) into the homes of older people: The REKI (REading KIng’s) research project. *Housing, care and support*. 2004;7(3):30-36. doi:10.1108/14608790200400022
83. Tsai HYS, Rikard RV, Cotten SR, Shillair R. Senior technology exploration, learning, and acceptance (STELA) model: from exploration to use - a longitudinal randomized controlled trial. *Educational gerontology*. 2019;45(12):728-743. doi:10.1080/03601277.2019.1690802
84. van Leersum CM, Konrad KE, Siebrand E, Malik ZB, den Ouden MEM, Bults M. Engaging older adults with a migration background to explore the usage of digital technologies in coping with dementia. *Frontiers in public health*. 2023;11:1125834-1125834. doi:10.3389/fpubh.2023.1125834
85. Velazquez A, Martinez-Garcia AI, Favela J, Hernandez A, Ochoa SF. Design of exergames with the collaborative participation of older adults. In: *Proceedings of the 2013 IEEE 17th International Conference on Computer Supported Cooperative Work in Design (CSCWD)*. IEEE; 2013:521-526. doi:10.1109/CSCWD.2013.6581016
86. Warner L, Tipping L. Can Everyday Assistive Technologies Provide Meaningful Support to Persons With Dementia and Their Informal Caregivers? Evaluation of Collaborative Community Program. *Journal of applied gerontology*. 2022;41(9):2022-2032. doi:10.1177/07334648221101041
87. Williams K, Pennathur P, Bossen A, Gloeckner A. Adapting Telemonitoring Technology Use for Older Adults: A Pilot Study. *Research in gerontological nursing*. 2016;9(1):17-23. doi:10.3928/19404921-20150522-01
88. Wu C, Hu X. A Study on the Behavior of Using Intelligent Television Among the Elderly in New Urban Areas. In: *Human Aspects of IT for the Aged Population. Acceptance, Communication and Participation*. Springer International Publishing; :194-205. doi:10.1007/978-3-319-92034-4_15
89. Yamazaki R, Nishio S, Nagata Y, et al. Long-term effect of the absence of a companion robot on older adults: A preliminary pilot study. *Frontiers in computer science (Lausanne)*. 2023;5. doi:10.3389/fcomp.2023.1129506
90. Yücel Y, Rizvanoğlu K. I am old too!: Understanding the Impact of Empathy and Voice Characteristics on Older Adults' Perception of Voice Assistants. In2022 IEEE International Conference on e-Business Engineering (ICEBE) 2022 Oct 14 (pp. 299-304). IEEE.
91. Zheng H, Rosal MC, Li W, et al. A Web-Based Treatment Decision Support Tool for Patients With Advanced Knee Arthritis: Evaluation of User Interface and Content Design. *JMIR human factors*. 2018;5(2):e17-e17. doi:10.2196/humanfactors.8568
92. Zhou J, Salvendy G. Senior Citizens Usage Towards and Perception of Modern Technology in India. In: *Human Aspects of IT for the Aged Population. Acceptance, Communication and Participation*. Vol 10926. Springer International Publishing AG; 2018.
93. Zhou P, Xie Y, Liang C. How to increase consumers’ continued use intention of artificial intelligence voice assistants? The role of anthropomorphic features. *Electronic markets*. 2023;33(1). doi:10.1007/s12525-023-00681-0
94. Zhu X. Learning and daily life integration: a qualitative analysis of the behaviors, characteristics, and logic of mobile learning among older adults. *Educational gerontology*. 2022;48(1):13-28. doi:10.1080/03601277.2021.2015677

1. **Voice activated virtual assistants** are devices with integrated systems like Alexa, Google Assistant, or Siri that offer full assistant functionalities, whereas others might simply respond to direct commands related to their operation. The former would be included, the latter would not be included. [↑](#footnote-ref-2)
2. **Wearables** are any smart technology that can be attached to a person and worn anywhere on the body that are constantly available for interaction with the user. Wearables typically have internet connectivity, include sensors, and are designed for interaction with the user. [↑](#footnote-ref-3)
